# TRIP6 enhances stemness property of breast cancer cells through activation of Wnt/β-catenin

**DOI:** 10.1186/s12935-020-1136-z

**Published:** 2020-02-14

**Authors:** Xiaohui Zhao, Chao Jiang, Rui Xu, Qingnan Liu, Guanglin Liu, Yan Zhang

**Affiliations:** 1 GMU-Joint School of Life Sciences, Guangzhou Medical University, Guangzhou, 511436 China; 2 Department of Cancer Center, People’s Hospital of Baoan District, Shenzhen, 518101 China; 3 Affiliated Cancer Hospital & Institute of Guangzhou Medical University, Guangzhou, 510095 China; 4 Novartis Oncology (China) AG, Guangzhou, 510630 China; 5 The First Affiliation Hospital of Guangzhou Medical University, Guangzhou, 510120 China; 6 Department of Medicine Oncology, Guangdong Provincial Key Laboratory of Colorectal and Pelvic Floor Diseases, The Sixth Affiliated Hospital, Sun Yat-sen University, Guangzhou, 510655 Guangdong China

**Keywords:** Breast cancer, Cancer stem cells, TRIP6, Prognosis, Wnt/β-catenin

## Abstract

**Background:**

The urgent problem in the treatment of breast cancer is the recurrence induced by breast cancer stem cells (CSCs). Understanding the role and molecular mechanism of specific molecules in breast cancer stem cells can provide a theoretical basis for better treatment. TRIP6 is an adapter protein which belongs to the zyxin family of LIM proteins and is important in regulating the functions of CSCs. The present study aims to investigate the effects and mechanism of TRIP6 in breast cancer.

**Methods:**

TRIP6 expression in breast cancer cells and tissues were detected by Real-Time PCR, western blot and immunohistochemistry (IHC). MTT assays, colony formation assays, Xenografted tumor model and mammosphere formation assays were performed to investigate the oncogenic functions of TRIP6 in the tumorigenic capability and the tumor-initiating cell-like phenotype of breast cancer cells in vitro and in vivo. Luciferase reporter, subcellular fractionation and immunofluorescence staining assays were performed to determine the underlying mechanism of TRIP6-mediated stemness of breast cancer cells.

**Results:**

TRIP6 expression was significantly upregulated in breast cancer, and was closely related to the clinicopathologic characteristics, poor overall survival (OS), relapse-free survival (RFS) and poor prognosis of breast cancer patients. Functional studies revealed that overexpression of TRIP6 significantly enhanced proliferative, tumorigenicity capability and the cancer stem cell-like properties of breast cancer in vitro and in vivo. On the contrary, silencing TRIP6 achieved the opposite results. Notably, we found that TRIP6 promoted Wnt/β-catenin signaling pathway in breast cancer to strengthen the tumor-initiating cell-like phenotype of breast cancer cells.

**Conclusions:**

This study indicates that TRIP6 plays an important role in maintaining the stem cell-like characteristics of breast cancer cells, supporting the significance of TRIP6 as a novel potential prognostic biomarker and therapeutic target for diagnosis and treatment of breast cancer.

## Background

Breast cancer is the most frequent cancer in women and one of the most common causes of cancer-related deaths worldwide [1]. Previous literature showed that about 30% to 50% of breast cancer patients were diagnosed at an early stage and could receive surgery, chemotherapy, radiotherapy, endocrine therapy or targeted therapy to improve curative potential, however beyond that, less than 10% of breast cancer patients would be newly diagnosed at advanced or metastatic stage, in which it is often very hard to cure the disease [2]. Despite much advance has been made in the treatment of breast cancer over the past decades, there is still about 40% of breast cancer patients have a recurrence and 60% of recurrent patients have metastasis, which makes the relapse of breast cancer remains to be the main obstacle to the complete cure of breast cancer patients [3]. Emerging evidence suggests that breast cancer initiation and maintenance may be regulated by a small population of cells within the tumor, which was named cancer stem cells (CSCs) [4]. CSCs have stronger tumorigenicity, metastatic ability and tumor-initiating properties in a variety of cancers, which leads to the tolerance to radiotherapy and chemotherapy [5–7]. Therefore, it is of great significance to explore effective tumor molecular markers and key regulatory mechanisms in CSCs.

Thyroid hormone receptor interacting protein 6 (TRIP6), an adapter protein, can interact with many different proteins through its LIM domain and act as an intracellular signal protein, transcriptional adapter or auxiliary activator [8]. Previous studies have shown that TRIP6 is an important auxiliary transcriptional activator which can be activated by transcriptional activating factor v-rel, therefore, TRIP6 can be used as a potential target to block v-rel-mediated tumorigenesis and transcriptional activation [9]. TRIP6 expression is up-regulated in glioma cells and tissues and its overexpression is correlated with poor clinical outcomes of glioma patients, which indicates that TRIP6 may become a novel prognostic biomarker and therapeutic target of glioma [10]. In addition, TRIP6 plays an important role in promoting the proliferation of hepatocellular carcinoma by increasing the activity of AKT and inhibiting the activity of FOXO3a and may become a new prognostic biomarker and therapeutic target of hepatocellular carcinoma [11]. However, how is the ability of TRIP6 to induce self-renewal of cancer cells and what is the precise molecular mechanism? These answers remain unknown.

Herein, we reported that TRIP6 is overexpressed in breast cancer and enhances cell proliferation and tumorigenicity abilities and tumor-initiating abilities of cancer stem cells. The mechanism may be that TRIP6 can promote Wnt/β-catenin pathway activation. These results provide a new insight into the role of TRIP6 in breast cancer.

## Materials and methods

### Patients and tissue samples

We collected complete clinical data of 340 breast cancer patients. All these patients were histopathologically and clinically diagnosed with breast cancer and accepted surgical operation and adjuvant chemotherapy at the first affiliation hospital of Guangzhou medical university from 2006 to 2011. Clinicopathological information about the samples was obtained retrospectively by analyzing patients’ files and summarized in Additional file 3: Table S1.

### Cell lines

Breast cancer cell lines, including ZR-75-30, T47D, MDA-MB-231, MDA-MB-415, and BT549 were cultured in Dulbecco’s Modified Eagle’s Medium (Cat#11965092, Gibco, Waltham, MA, USA) supplemented with 10% FBS (Cat#SH30084.03, HyClone, Pittsburgh, PA, USA); and immortal human mammary epithelial cell line (MCF-10a) was maintained in MEGM™ mammary epithelial cell growth medium bulletKit™ (Cat#LONZA-192853, Lonza, Basel, Switzerland).

### RNA extraction and Real-time PCR

Total RNA from MCF-10a and breast cancer cell lines and primary breast cancer samples was extracted using the Trizol reagent (Cat#15596026, Invitrogen, Carlsbad, CA, USA) according to the manufacturer’s instruction. cDNAs were amplified and quantified in ABI Prism 7500 Sequence Detection System (Thermo Fisher Scientific) using dye SYBR Green (Thermo Fisher Scientific). The primers were selected as the following: *TRIP6* (forward primer: 5′-TCGAAGTTTCCACATCGGCT-3′, reverse primer 5′-GCTCTTGGATACGCCAGGC-3′) [12]; expression data were normalized to the geometric mean of housekeeping gene *GAPDH* (forward: 5′-ACCACAGTCCATGCCATCAC-3′ and reverse: 5′-TCCACCACCCTGTTGCTGTA-3′) to control the variability in expression levels and calculated as 2−^[(Ct of gene) − (Ct of GAPDH)]^, where Ct represents the threshold cycle for each transcript.

### Vectors and retroviral infection

The human TRIP6 gene was PCR-amplified from cDNA and cloned into a pSin-EF2 lentiviral vector. To silence TRIP6, a TRIP6-targeting short hairpin RNA (shRNA) sequence was cloned into a SUPER.retro.puro vector (OligoEngine, Washington, USA) to generate the respective pSUPER.retro.TRIP6-RNAi(s). The targeting sequence is 5′-GAAGCTGGTTCACGACATGAA-3′ [13]. Retroviral production and infection were performed as previously described [14]. Stable cell lines expressing TRIP6 or TRIP6 shRNAs were selected for 10 days with 0.5 μg/ml puromycin. The TOP Flash and FOP Flash reporters containing the wild-type and mutated TCF/LEF DNA-binding sites, respectively, were purchased from Upstate Biotechnology (Lake Placid, NY, USA). Transfection of siRNAs (Ribo Biotech, Guangzhou) or psin-EF2-TRIP6 and pSUPER. retro. TRIP6-RNAi plasmids (5 μg) were performed using the Lipofectamine 2000 reagent (Cat#11668019, Invitrogen, Carlsbad, CA, USA).

### Western blot

Western blot was performed according to standard methods as described previously [15], using anti-TRIP6 antibody (1:1000, Cat#ab137478, Abcam, Cambridge, UK), anti-GSK3β (1:1000, Cat#12456, Cell signaling technology, Danvers, MA, USA), anti-p-GSK3β (Ser9) (1:1000, Cat#5558, Cell signaling technology, Danvers, MA, USA), anti-p-β-catenin (Y142) (1:500, Cat#ab27798, abcam, Cambridge, UK), anti-β-catenin (1:1000, Cat#9562, Cell signaling technology, Danvers, MA, USA) antibody, anti-P84 (1 μg/ml, Cat#ab487, Abcam, Cambridge, UK) antibody, anti-GAPDH antibody (1:2000, Cat#G9545-200UL, Sigma-Aldrich, Saint Louis, MO, USA) was used as a loading control.

### MTT cell viability assay

Cells were seeded in 96-well plates at a density of 2 × 10^3^ cells/well. At each time point, cells were stained with 100 μl sterile MTT dye (0.5 mg/ml, Cat#M2003, Sigma-Aldrich, Saint Louis, MO, USA) for 4 h at 37 °C, followed by removal of the culture medium and addition of 100 μl of dimethyl sulphoxide (Cat#D8418, Sigma-Aldrich, Saint Louis, MO, USA). The absorbance was measured at 490 nm wavelength. Each experiment was performed in triplicates.

### Colony formation assay

Cells were plated in 6-well plates (5 × 10^2^ cells) and cultured for 10 days. The colonies were stained with 1% crystal violet for 30 s after fixation with 4% formaldehyde for 5 min. After washing with PBS, the number of colonies (> 50 cells/colony) was counted with a microscope.

### Xenografted tumor model

Female BALB/c nude mice (5–6 weeks of age, 18–20 g) were purchased from the Slaca-Jingda Laboratory Animal (Hunan, China) and house them in barrier facilities on a 12-h light/dark cycle. All experimental procedures were approved by the Institutional Animal Care and Use Committee of Guangzhou Medical University. All mice were randomly divided into 4 groups (n = 5/group). 200 µl 2 × 10^6^ ZR-75-30/TRIP6 and ZR-75-30/shTRIP6 stably breast cancer cells were injected into the mammary fat pad of mice. Tumors were measured twice a week, the length, width, and thickness of the tumors were measured with calipers, and tumor volume was calculated using the equation (*L* × *W*^2^)/2. On day 27, the mice were euthanized, and tumors were excised and weighed.

### Mammosphere formation assays

Five hundred cells were seeded in 6-well ultra-low cluster plates and 100 cells were seeded in 24-well ultra-low cluster plates for 10 days. Spheres were cultured in DMEM/F12 serum-free medium (Cat#11330-032, Invitrogen, Carlsbad, CA, USA) supplemented with 2% B27 (Cat#17504044, Invitrogen, Carlsbad, CA, USA), 20 ng/ml EGF (Cat#AF-100-15, PeproTech, Rocky Hill, NJ, USA), 20 ng/ml bFGF (Cat#AF-100-18C-50, PeproTech, Rocky Hill, NJ, USA), 0.4% BSA (Cat#A1933, Sigma-Aldrich, St. Louis, MO, USA), and 5 μg/ml insulin. After 10 days, the tumor spheres (tight, spherical, non-adherent masses > 50 μm in diameter) were counted, and their images were captured under an inverted microscope.

### Luciferase reporter assay

Cells (1 × 10^4^) were seeded in triplicate in 48-well plates and allowed to settle for 24 h. Then, 100 ng of the indicated plasmids plus 1 ng of the pRL-TK Renilla plasmid was transfected into cells using Lipofectamine 2000 reagent (Cat#11668019, Invitrogen, Carlsbad, CA, USA) according to the manufacturer’s instruction. 48 h after transfection, luciferase and Renilla signals were measured using the Dual Luciferase Reporter Assay Kit according to the manufacturer’s instructions (Cat#E1910, Promega, Madison, WI, USA).

### Preparation of nuclear extracts

Confluent ZR-75-30 and MDA-MB-231 cells in T75 flasks were washed with 5 ml PBS/phosphatase inhibitors, the supernatant aspirated, and 3 ml ice-cold PBS/phosphatase inhibitors added. The cells were removed by gently scraping with a cell lifter and transferred to a pre-chilled 15 ml conical tube. The cell suspension was centrifuged for 5 min at 200×*g* in a centrifuge precooled at 4 °C, and the supernatant was discarded. A Nuclear Extract Kit (Cat#40010, Active Motif, Rixensart, Belgium) was used according to the manufacturer’s instructions for isolating nuclear extracts from cell pellets.

### Immunofluorescence assay

Cells were seeded on coverslips (Thermo Fisher Scientific, Lafayette, CO) and cultured in 24-well plate overnight. Immunostaining was performed according to a previous report [16]. Anti-β-catenin (1:200, Cat#9562, Cell signaling technology, Danvers, MA, USA) antibody and species-specific FITC-conjugated secondary antibodies (1:500; Invitrogen, Carlsbad, CA, USA) were used. Image-Pro Plus 6.0 (Olympus) was used for image analysis.

### Statistical analysis

All statistical analyses were carried out using the SPSS 19.0 statistical software package. The Chi square test was used to analyze the relationship between TRIP6 expression and clinicopathological characteristics. Bivariate correlations between study variables were calculated by Spearman correlation analysis. Overall Survival curve and relapse-free survival curve were plotted by the Kaplan–Meier method and compared using the log-rank test. Survival rates were evaluated using univariate and multivariate Cox regression analyses. *P* < 0.05 was considered statistically significant.

## Results

### Elevated TRIP6 expression is associated with poor prognosis in human breast cancer patients

**Fig. 1:**
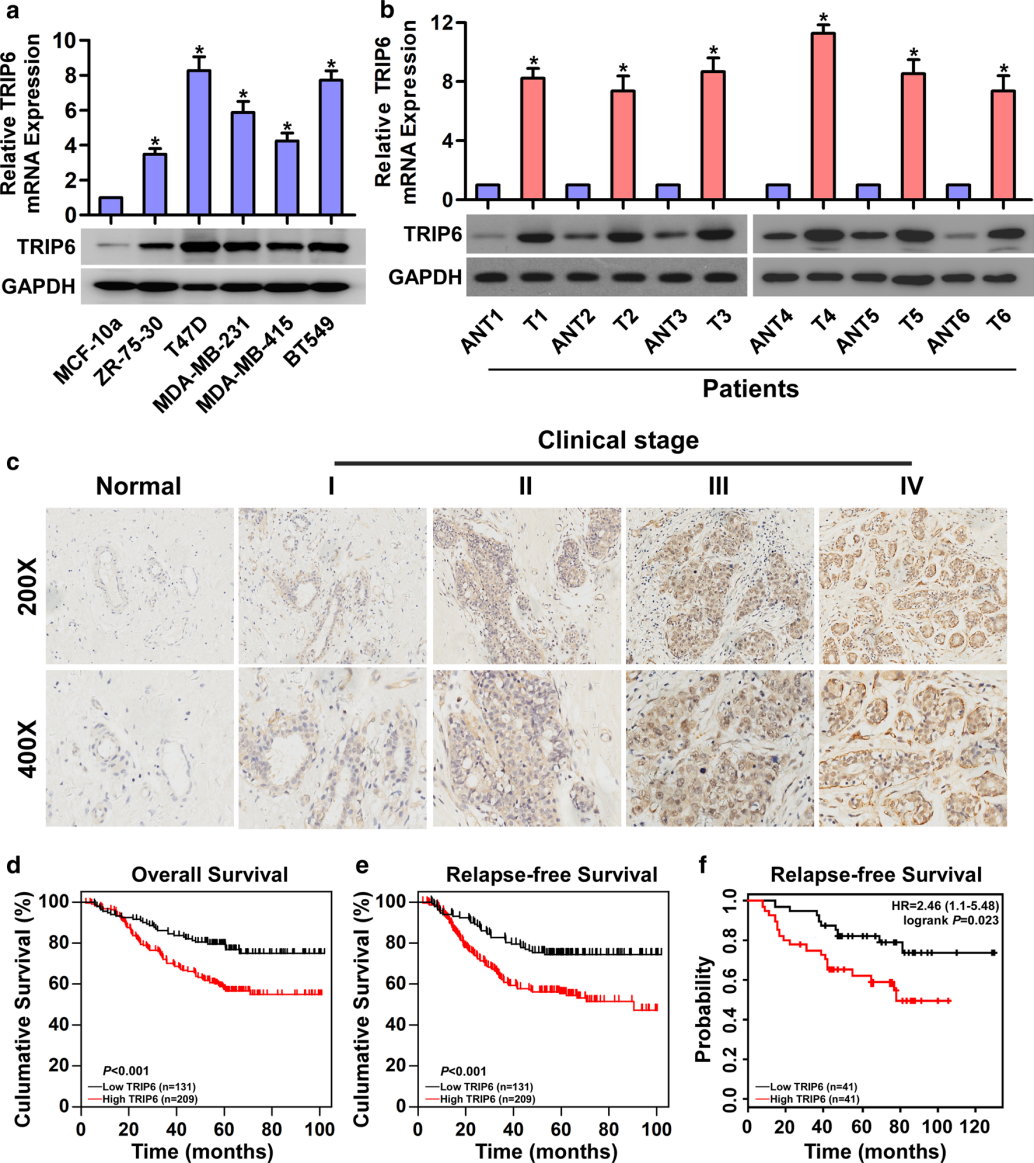
Elevated TRIP6 expression is associated with poor prognosis in human breast cancer patients. **a** Real-time PCR and western blot analysis of TRIP6 expression in immortalized human breast epithelial cell line (MCF-10a) and five breast cancer cell lines. **b** Real-time PCR and western blot analysis of TRIP6 expression in six matched breast cancer tissues (T) and adjacent noncancerous tissues (ANT). TRIP6 mRNA levels were normalized to that of *GAPDH*. TRIP6 protein levels were normalized to GAPDH. **c** The expression of TRIP6 in normal breast tissues and breast cancer tissues at different clinical stages. **d** Kaplan–Meier survival analysis of the correlation between TRIP6 expression level and 5-year OS in breast cancer patients. **e** Relapse-free survival (RFS) of breast cancer patients with low versus high TRIP6 expression. **f** Kaplan–Meier analysis of the RFS in breast cancer using Kaplan–Meier Plotter (http://kmplot.com/analysis/)

To determine the potential effect of TRIP6 in breast cancer, we first validated TRIP6 mRNA and protein expression level in six breast cell lines (MCF-10a, ZR-75-30, T47D, MDA-MB-231, MDA-MB-415, and BT549). As shown in Fig. 1a, TRIP6 mRNA and protein expression were significantly elevated in five breast cancer cell lines compared to immortal human mammary epithelial cell line (MCF-10a). Simultaneously, we explored the NCBI/GEO database (GSE42568) (https://www.ncbi.nlm.nih.gov/gds/?term=) to investigate the expression of TRIP6 in breast cancer. Consistently, compared with normal tissues, TRIP6 mRNA expression was significantly up-regulated in breast tumors (Additional file 1: Fig. S1). Moreover, our results also confirmed that TRIP6 was overexpressed in six breast cancer tissues compared with corresponding adjacent noncancerous tissues (Fig. 1b and Additional file 2: Fig. S2). These results demonstrated that TRIP6 is upregulated in breast cancer.

To explore the relationship between TRIP6 expression and the clinicopathological type of breast cancer patients, we detected the expression of TRIP6 protein in 340 paraffin-embedded breast cancer tissues (Additional file 3: Table S1). The results showed that with the increase of breast cancer clinical stage, the TRIP6 expression level was also upregulated in the tissues (Fig. 1c). Statistical analysis further revealed that TRIP6 expression was significantly correlated with the clinical stage (*P *< 0.001), T classification (*P *= 0.019), M classification (*P *= 0.046) and relapse (*P *= 0.001) of breast cancer patients (Additional file 3: Table S2). Kaplan–Meier survival curves and the log-rank test showed that TRIP6 expression was significantly negatively correlated with the OS and RFS of breast cancer patients (*P* < 0.01; Fig. 1d, e). Assessment from the publicly available KM Plotter database (http://kmplot.com/analysis/index.php?p=service&cancer=breast) showed that high TRIP6 expression correlated with worse RFS of breast cancer patients (Fig. 1f), which is consistent with and further validated our results. Moreover, univariate and multivariate analyses indicated that TRIP6 expression was an independent prognostic factor for the breast cancer patients (Additional file 3: Table S3), which suggests that TRIP6 may represent a novel prognostic biomarker for breast cancer.

### TRIP6 promotes the proliferation and tumorigenicity of breast cancer cells

**Fig. 2:**
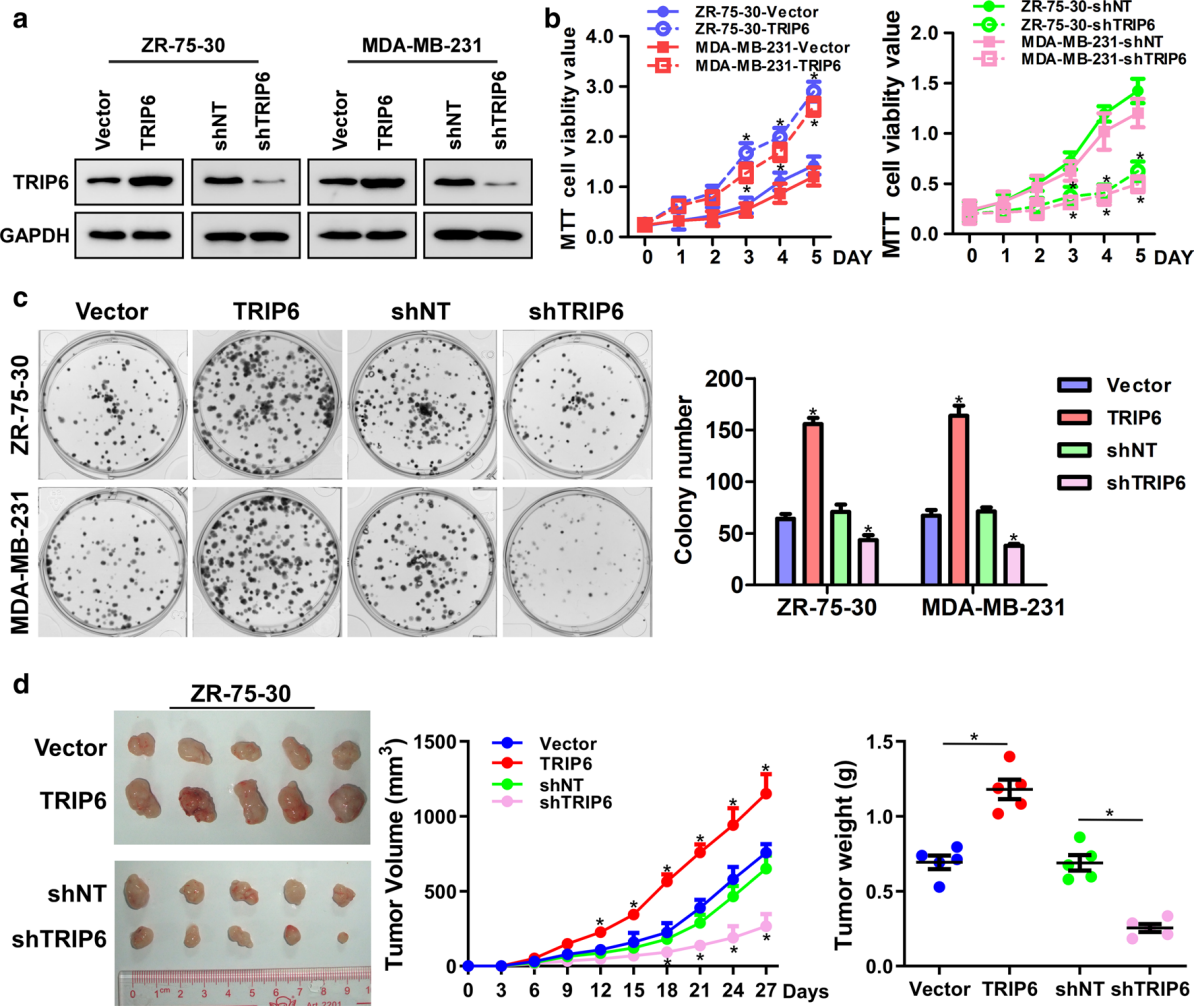
TRIP6 promotes the proliferation and tumorigenicity of breast cancer cells. **a** Confirmation of the overexpression and knockdown of TRIP6 in ZR-75-30 and MDA-MB-231 cells; GAPDH was used as a loading control. **b**, **c** MTT and colony formation assays indicated that the growth rates increased in TRIP6-transduced breast cancer cells and decreased in TRIP6-silenced breast cancer cells. The number of colonies was quantified in the colony formation assay. Error bars represent the mean ± SD of three independent experiments. *P < 0.05. **d** ZR-75-30 cells with overexpression of TRIP6 and silencing TRIP6 were inoculated into the fat pad (n = 5/group) of mice. A representative image of each group of mouse tumors (left). Indicates the tumor volume growth curve (center) and average tumor weight (right) of the tumor formed by the indicated cells. Error bars represents the mean ± SD. *P < 0.01

Because the results from the clinical specimens indicate that there is a correlation between TRIP6 expression and clinical stage and T classification, so we used stable TRIP6-transduced and TRIP6-silenced cells to further investigate the effect of TRIP6 on the proliferation of breast cancer cells (Fig. 2a). MTT and colony formation assays indicated that the proliferation rate was significantly increased in TRIP6-transduced breast cancer cells compared with the vector cells, and conversely decreased in TRIP6-silenced breast cancer cells (Fig. 2b, c). Furthermore, we studied whether TRIP6 could promote the tumorigenicity of breast cancer cells in vivo. The growth rate of tumors formed by ZR-75-30/TRIP6 was significantly faster than that of vector control cells at all time points, while the growth rate of tumors formed by ZR-75-30/shTRIP6 cells was significantly slower than that of shNT control cells (*P* < 0.01; Fig. 2d). In addition, breast cancer cells transduced with TRIP6 formed larger tumors and had higher tumor weights than vector control tumors. On the contrary, the tumors formed by TRIP6 silencing cells were smaller than that formed by shNT control cells both in size and weight. Collectively, our results provided strong evidence that TRIP6 plays a vital role in promoting cell proliferation and tumorigenicity of breast cancer cells.

### TRIP6 enhances the stemness of breast cancer cells

**Fig. 3:**
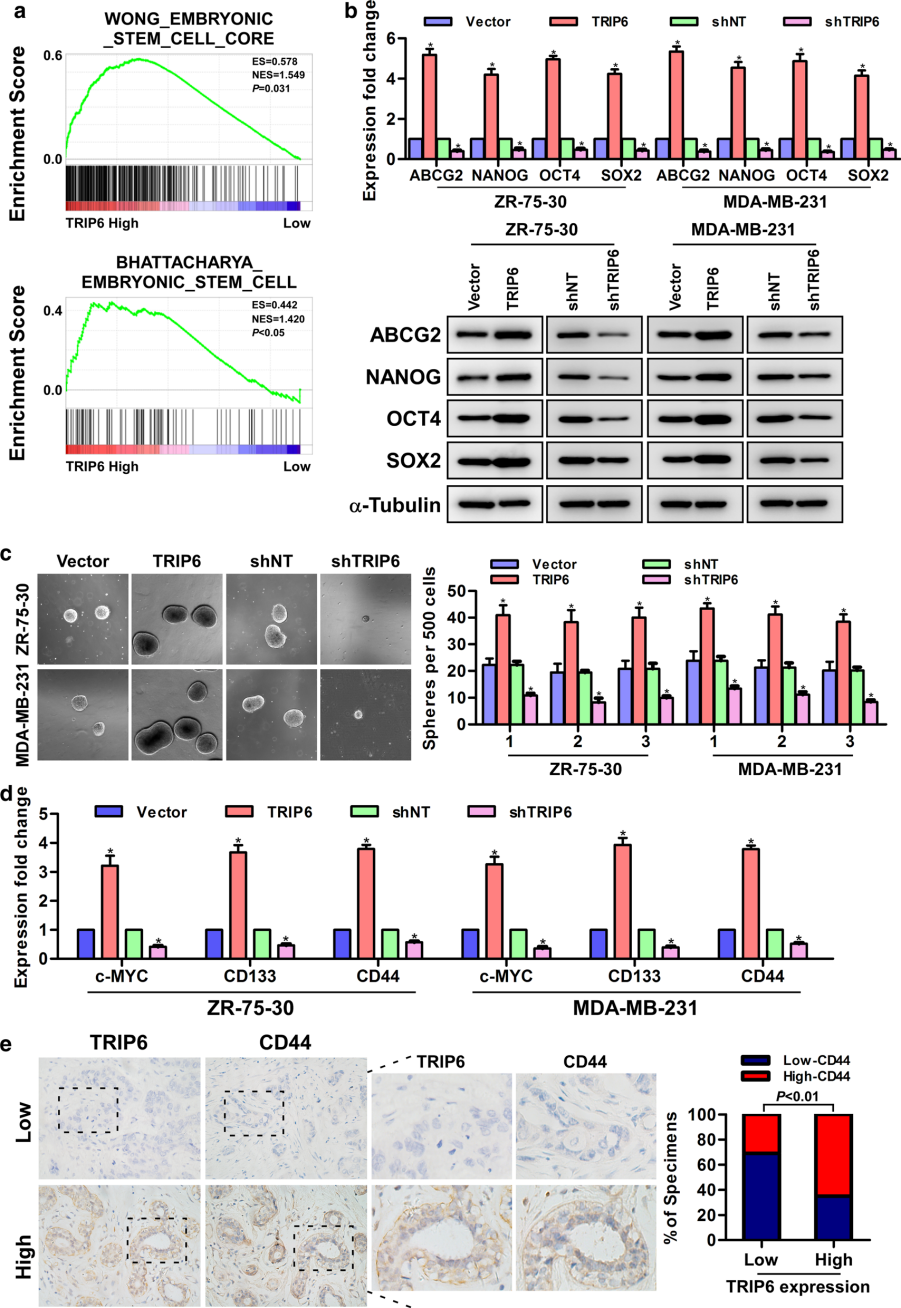
TRIP6 enhances the stemness of breast cancer cells. **a** GSEA indicated significant correlations between TRIP6 mRNA expression and stemness-related gene signatures (WONG_EMBRYONIC_STEM_CELL, BHATTACHARYA_ EMBRYONIC_ STEM_CELL). **b** Real-time PCR and Western blot analysis of stemness-related markers in the indicated cells. Error bars represent the mean ± SD of three independent experiments; *P < 0.05. **c** Representative micrographs (left) and quantification (right) of mammosphere formation by TRIP6-transduced cells, TRIP6-silenced cells and vector cells. Error bars represent the mean ± SD of three independent experiments; *P < 0.05. **d** Real-time PCR of the mRNA expression of cancer stem cell markers. Error bars indicate the mean ± SD of three independent experiments; *P < 0.05. **e** TRIP6 expression levels significantly correlated with CD44 expression in breast cancer tissues (n = 340; *P *< 0.01). Two representative cases are shown (left) and percentage of specimens with low or high TRIP6 expression, relative to the levels of CD44 staining (right)

As shown in Fig. 1e, f, overexpression of TRIP6 is significantly associated with the relapse-free survival of breast cancer patients, and besides that, previous studies have shown that cancer stem cells (CSCs) play a key role in tumor recurrence [17], which suggests that TRIP6 may be involved in the regulation of stemness. Consistently, Gene set enrichment analysis (GSEA) of a publicly available GEO database (GSE42568) revealed that TRIP6 expression was positively associated with stemness signatures (WONG_EMBRYONIC_STEM_CELL, BHATTACHARYA_ EMBRYONIC_ STEM_CELL) (Fig. 3a). In addition, our real-time PCR and Western blot results revealed that the mRNA and protein expression levels of stemness-related markers, including ABCG2, NANOG, OCT4 and SOX2, were significantly increased in TRIP6-transduced ZR-75-30 and MDA-MB-231 cells, and conversely downregulated in TRIP6-silenced breast cancer cells (Fig. 3b). Next, we conducted the mammosphere formation assay to detect the effect of TRIP6 on the self-renewal ability of breast cancer cells. Notably, the results showed that TRIP6-transduced breast cancer cells formed larger and more spheres than vector cells, whereas TRIP6-silenced cells formed smaller and fewer spheres than shNT control cells (Fig. 3c). In addition, we detected the mRNA expression of cancer stem cell makers, including c-MYC, CD44 and CD133, and found that their expression were increased in TRIP6-transduced ZR-75-30 and MDA-MB-231 cells but were decreased in TRIP6-silenced cells (Fig. 3d). Furthermore, we used immunostaining assay to detect the correlation between the expression of TRIP6 and CD44 in breast cancer patient specimens, and found that in the breast cancer samples, the areas that displayed high levels of TRIP6 staining also showed strong CD44 staining, while areas with low TRIP6 expression also displayed weakly detectable CD44 expression (Fig. 3e), indicating that the expression of TRIP6 was significantly correlated with the expression of cancer stem cell marker-CD44. Taken together, our results showed that TRIP6 promotes the stem-like phenotype of breast cancer cells.

### Mechanism of TRIP6-mediated expansion of cancer stem-like cells

**Fig. 4:**
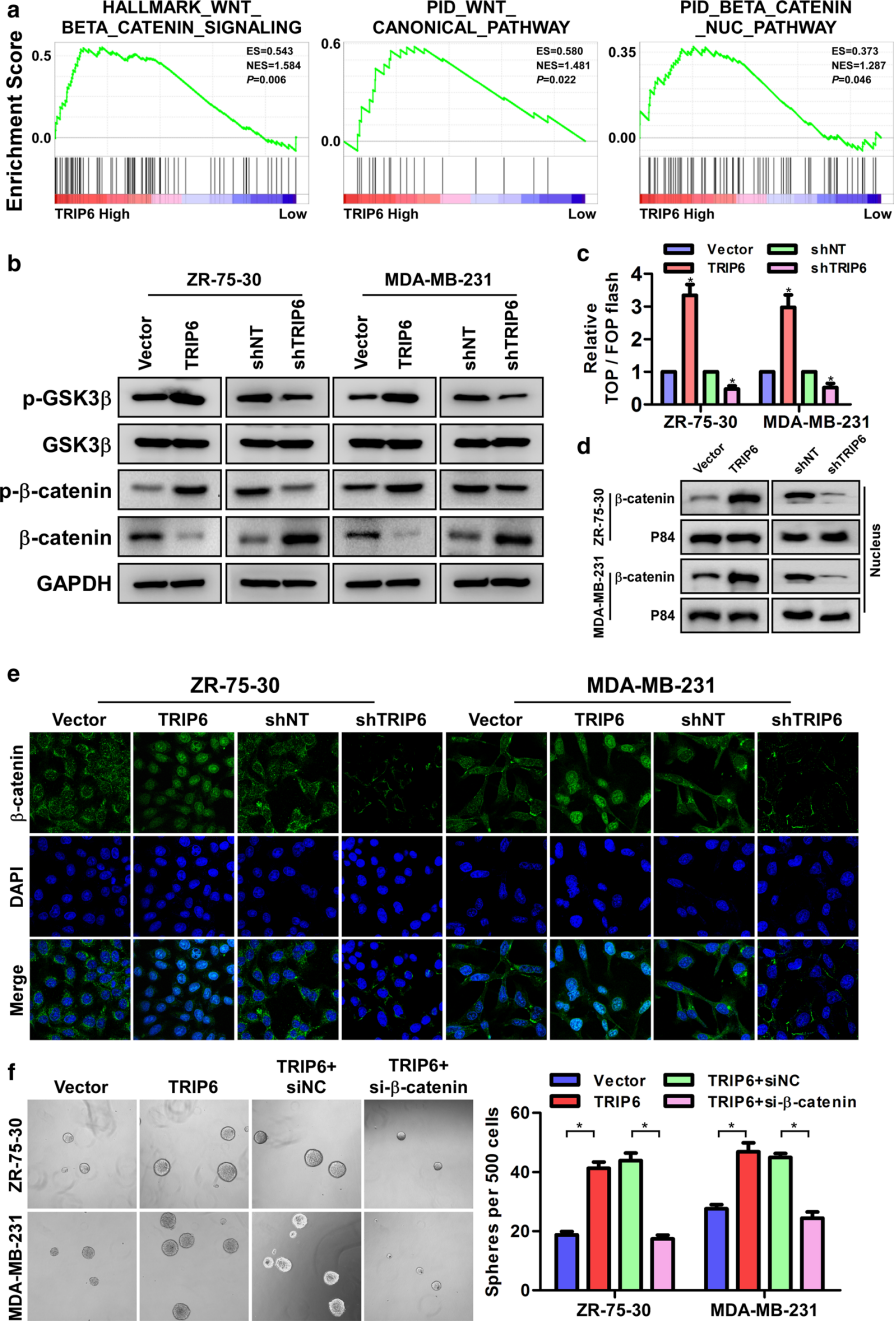
Mechanism of TRIP6-mediated expansion of cancer stem-like cells. **a** GSEA showed the positive correlations between TRIP6 mRNA levels and Wnt/β-catenin-activated gene signatures (HALLMARK_WNT_BETA_CATENIN_SIGNALING and PID_WNT_CANONICAL_PATHWAY) and β-catenin nucleus gene signature (PID_BETA_CATENIN_NUC_PATHWAY). **b** The cellular protein levels of p-GSK3β (Ser9), GSK3β, p-β-catenin (Y142) and β-catenin in indicated ZR-75-30 and MDA-MB-231 cells were detected by Western blotting assay. GAPDH was used as the cellular control. **c** Luciferase-reporter assays of TOP/FOP transcriptional activity in the indicated cells. Error bars represent the mean ± SD of three independent experiments; *P < 0.05. **d** Western blot analysis of β-catenin in the nuclear fractions of the indicated cells. **e** Immunofluorescence staining showed the localization of β-catenin in the indicated cells. **f** Representative images and quantification of cellular spheres formed by the indicated cells. Error bars represent the mean ± SD of three independent experiments; *P < 0.05

To explore the mechanism of TRIP6-mediated stem-like characteristics in breast cancer, we next studied the relationship between TRIP6 expression and the genes regulated by various signaling signatures using GSEA of GEO datasets. GSEA results showed that TRIP6 expression positively correlated with Wnt/β-catenin-activated gene signatures (HALLMARK_WNT_BETA_CATENIN_SIGNALING and PID_WNT_CANONICAL_PATHWAY) and β-catenin nucleus gene signature (PID_BETA_CATENIN_NUC_PATHWAY) in gene expression profiles of breast cancer patients obtained from the GEO database (GSE42568) (Fig. 4a), indicating that TRIP6 may activate the Wnt/β-catenin signaling pathway. Western blot analysis showed that the protein levels of p-GSK3β (Ser9) and p-β-catenin (Y142) in TRIP6-transduced ZR-75-30 and MDA-MB-231 cells were significantly increased, on the contrary, the protein levels of p-GSK3β (Ser9) and p-β-catenin (Y142) in silenced-TRIP6 cells were significantly decreased (Fig. 4b). Furthermore, TOP/FOP flash assays revealed that overexpression of TRIP6 markedly increased whereas TRIP6 knockdown attenuated the transcriptional activation of TCF/LEF in the ZR-75-30 and MDA-MB-231 cells (Fig. 4c). Meanwhile, subcellular fractionation and immunofluorescence staining assays showed that overexpression of TRIP6 substantially increased, whereas knockdown of TRIP6 reduced the nuclear signals of β-catenin (Fig. 4d, e), indicating that TRIP6 promotes the translocation of β-catenin into the nucleus by activating the GSK3β signaling pathway. Next, we further examined the role of Wnt/β-catenin activation in TRIP6-induced stemness. As shown in Fig. 4f, silencing β-catenin in TRIP6-tranduced cells strikingly reversed the spheroidizing ability of breast cancer cells (Fig. 4f). The mRNA levels of the downstream targets of Wnt/β-catenin signaling, including c-MYC, TCF1, CD133 and CD44, were increased in TRIP6-transduced ZR-75-30 and MDA-MB-231 cells but were decreased in TRIP6-silenced cells (Additional file 4: Fig. S3). Taken together, our results suggested that upregulation of TRIP6 promotes the proliferation, tumorigenicity and stemness of breast cancer cells through enhancing Wnt/β-catenin signaling.

## Discussion

The main findings of this study are that TRIP6 is upregulated in breast cancer, TRIP6 is associated with poor overall survival and relapse-free survival of breast cancer patients and TRIP6 promotes proliferation and stemness of breast cancer cells through activating Wnt/β-catenin signaling pathway, suggesting that TRIP6 may be a valuable prognostic factor for the prognosis and recurrence of breast cancer patients.

Previous studies show that TRIP6 is a novel molecular partner which belongs to the family of LIM proteins and plays a crucial role in cell motility, actin cytoskeleton reorganization, transcriptional regulation and so on [18–21]. The single-stranded telomeric overhang binding protein POT1 is a component of the Shelterin complex that regulates telomere length. Nuclear TRIP6 binds to POT1 and protects the ends of chromosomes [22]. Depletion of TRIP6 leads to the occurrence of telomere dysfunction-induced foci [22]. TRIP6 is significantly overexpressed in glioblastomas with aberrantly elevated c-src activation and is related to the progress of glioblastomas [23]. In our study, we found that TRIP6 is upregulated in breast cancer cell lines and tissues. Moreover, statistical analysis revealed that high TRIP6 expression is correlated with poor prognosis, short overall survival and relapse-free survival of breast cancer patients, indicating that TRIP6 may represent a potentially useful prognostic biomarker for breast cancer patients.

The ectopic expression of TRIP6 could decrease cell–cell aggregation and promote invasiveness of the epithelial Madin-Darby Canine Kidney (MDCK) and MDCKts-src cells by enhancing the activation of NF-κB signaling [24]. Besides, TRIP6 could promote lysophosphatidic acid (LPA)-induced ovarian cancer cell migration by activating AKT signaling [25]. In this study, the results of MTT, colony formation and tumorigenicity showed that TRIP6 promoted the proliferation and tumorigenesis of breast cancer cells in vitro and in vivo. In addition, mammosphere formation results indicated that TRIP6 overexpression enhanced the self-renewal ability of breast cancer stem cells, while silencing TRIP6 weakened the spheroidizing ability of breast cancer stem cells. Taken together, these data indicated that TRIP6 enhances proliferation, tumorigenesis and stemness of breast cancer cells, thus inducing the development and recurrence of breast cancer.

In breast cancer, it is reported that the population of cancer stem cells may be a source of chemotherapy resistance and tumor recurrence, and one of the mechanism is the activation of Wnt/β-catenin pathway, suggesting that Wnt/β-catenin signaling pathway may be involved in the resistance to current anticancer drugs and breast cancer recurrence by regulating the population of cancer stem cells [26–30]. Indeed, many studies have shown that Wnt/β-catenin signaling pathway may play a significant role in maintaining mammary stem cell properties [31–33]. In this study, we further investigated the molecular mechanism of Wnt/β-catenin signaling activation in breast cancer, hoping to provide a potentially valuable therapeutic target for preventing recurrence of breast cancer. Our results demonstrated that TRIP6 is not only involved in the activation of Wnt/β-catenin signaling pathway and regulating the expression of its downstream target gene, but also promotes the stem-like phenotype of breast cancer cells, indicating that TRIP6 is a driver molecule for breast cancer recurrence by activating the Wnt/β-catenin signaling.

## Conclusions

In conclusion, we demonstrated that TRIP6 is overexpressed in breast cancer cells and tissues and is associated with the poor prognosis of breast cancer patients. Also, we found that TRIP6 is involved in activating Wnt/β-catenin pathway and promoting the expression of its downstream gene, resulting in enhanced proliferation ability of breast cancer cells and self-renewal ability of CSCs. Therefore, TRIP6 is a potential novel prognostic biomarker and therapeutic target for recurrence of breast cancer.

## Supplementary information

**Additional file 1: Fig. S1.** Expression level of TRIP6 in normal (N) and tumor (T) tissues of patients from the GEO/GSE42568 dataset. Error bars represent Median with interquartile range.

**Additional file 2: Fig. S2.** Immunohistochemistry analysis of the expression of TRIP6 in paired adjacent non-tumor (NT) and tumor (T) tissues from breast cancer patients.

**Additional file 3: Table S1.** Clinicopathological characteristics of 340 patient samples and expression of TRIP6 in Breast cancer. **Table S2.** Correlation between TRIP6 expression and clinicopathologic characteristics of breast cancer. **Table S3**. Univariate and multivariate analyses of various prognostic parameters in patients with breast cancer Cox-regression analysis.

**Additional file 4: Fig. S3.** The mRNA levels of the downstream targets of Wnt/β-catenin signaling, including c-MYC, TCF1, CD133 and CD44 in indicated breast cancer cells.

## Data Availability

All data generated or analyzed during this study are included in this published article.

## Abbreviations

CSCs: Cancer stem cells
TRIP6: Thyroid hormone receptor interacting protein 6
IHC: Immunohistochemistry
MTT: 3-(4,5-dimethylthiazol-2-yl)-2,5-dimethyltetrazolium bromide
ABCG2: ATP binding cassette subfamily G member 2
NANOG: Nanog homeobox
OCT4: Organic cation/carnitine transporter 4
SOX2: Sex determining region Y box 2
c-Myc: MYC proto-oncogene
TCF1: Transcription factor T cell factor 1
MDCK: Madin-Darby Canine Kidney
LPA: Lysophosphatidic acid
GSEA: Gene set enrichment analysis
