# Supplementary figures and images for: TRIP6 enhances stemness property of breast cancer cells through activation of Wnt/β-catenin
Source: Cancer Cell Int. 2020 Feb 14;20:51. doi: 10.1186/s12935-020-1136-z (PMC7023708; doi:10.1186/s12935-020-1136-z)

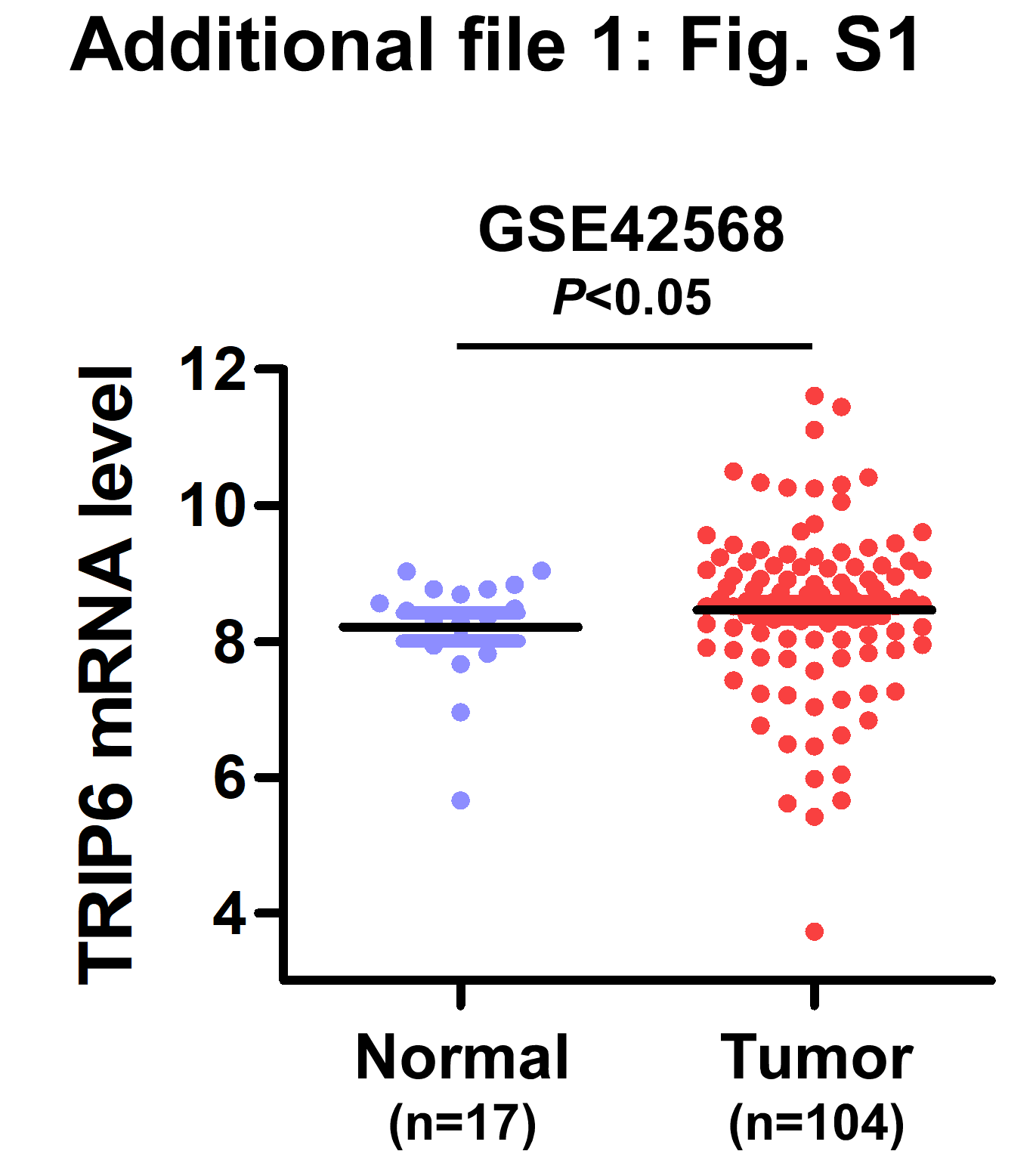

Supplement: Supplementary file 1 — Additional file 1: Fig. S1. Expression level of TRIP6 in normal (N) and tumor (T) tissues of patients from the GEO/GSE42568 dataset. Error bars represent Median with interquartile range. [file 12935_2020_1136_MOESM1_ESM.tif]

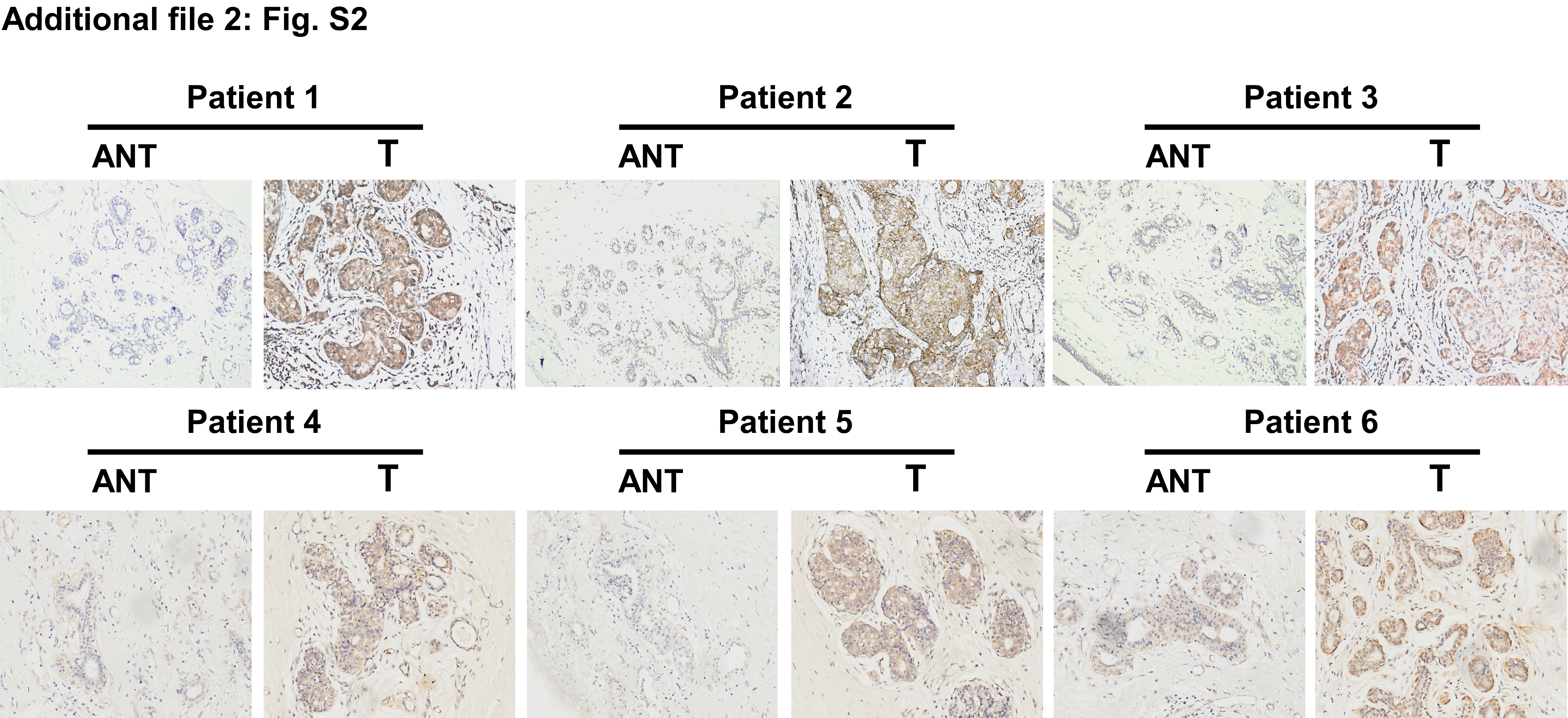

Supplement: Supplementary file 2 — Additional file 2: Fig. S2. Immunohistochemistry analysis of the expression of TRIP6 in paired adjacent non-tumor (NT) and tumor (T) tissues from breast cancer patients. [file 12935_2020_1136_MOESM2_ESM.tif]

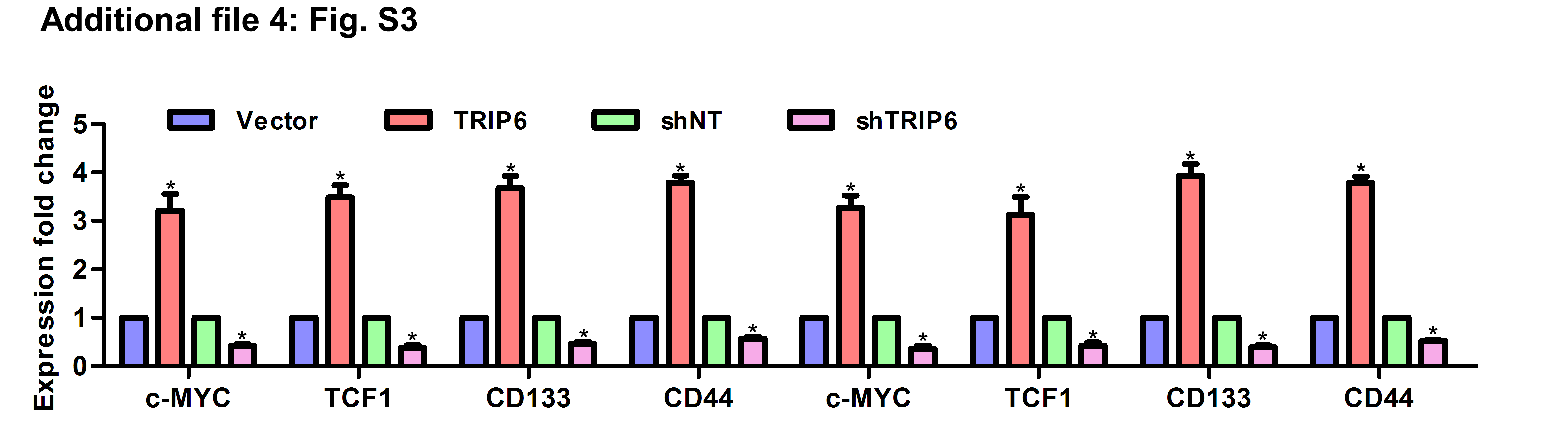

Supplement: Supplementary file 4 — Additional file 4: Fig. S3. The mRNA levels of the downstream targets of Wnt/β-catenin signaling, including c-MYC, TCF1, CD133 and CD44 in indicated breast cancer cells. [file 12935_2020_1136_MOESM4_ESM.tif]
