# Supplementary material for: TRIP6 enhances stemness property of breast cancer cells through activation of Wnt/β-catenin
Source: Cancer Cell Int. 2020 Feb 14;20:51. doi: 10.1186/s12935-020-1136-z (PMC7023708; doi:10.1186/s12935-020-1136-z)
Supplement: Supplementary file 3 — Additional file 3: Table S1. Clinicopathological characteristics of 340 patient samples and expression of TRIP6 in Breast cancer. Table S2. Correlation between TRIP6 expression and clinicopathologic characteristics of breast cancer. Table S3. Univariate and multivariate analyses of various prognostic parameters in patients with breast cancer Cox-regression analysis. [file 12935_2020_1136_MOESM3_ESM.docx]

Additional file 3

Table S1.

Clinicopathological characteristics of 340 patient samples and expression of TRIP6 in Breast cancer

| Characteristic | Subgroup | Number of cases (%) |
| --- | --- | --- |
| Age (years) | ≥ 52 | 217 (63.8%) |
|  | <52 | 123 (36.2%) |
| Clinical Stage | I | 40 (11.8%) |
|  | II | 163 (47.9%) |
|  | III | 99(29.1%) |
|  | IV | 38 (11.2%) |
| T classification | T1 | 74 (21.8%) |
|  | T2 | 172 (50.6%) |
|  | T3 | 67 (19.7%) |
|  | T4 | 27 (7.9%) |
| N classification | N0 | 145 (42.6%) |
|  | N1 | 125 (36.8%) |
|  | N2 | 56 (16.5%) |
|  | N3 | 14 (4.1%) |
| M classification | M0 | 302 (88.8%) |
|  | M1 | 38 (11.2%) |
| Tumor grade | Well differentiated | 90 (26.5%) |
|  | Moderately differentiated | 135 (39.7%) |
|  | Poorly differentiated | 80 (23.5%) |
|  | Unknown | 35 (10.3%) |
| Molecular subtype | Luminal A | 80 (23.5%) |
|  | Luminal B | 105 (30.9%) |
|  | TNBC | 90 (26.5%) |
|  | HER2(+) | 65 (19.1%) |
| Vital status (at follow-up) | Alive | 223 (65.6%) |
|  | Death (all breast cancer-related) | 117 (34.4%) |
| Relapse or metastasis | Yes | 125(36.8%) |
|  | No | 215(63.2%) |
| CD44 expression | Low expression | 167（49.1%） |
|  | High expression | 173（50.9%） |
| TRIP6 expression | Low expression | 131 (38.5%) |
|  | High expression | 209 (61.5%) |

Table S2.

Correlation between TRIP6 expression and clinicopathologic characteristics of breast cancer

| Characteristics | | TRIP6 | | Chi-square test *P*-value |
| --- | --- | --- | --- | --- |
|  |  | Low or none No. cases (%) | High  No. cases (%) |  |
| Age (years) | ≥ 52 | 78 (35.9) | 139 (64.1) | 0.193 |
|  | < 52 | 53 (43.1) | 70 (56.9) |  |
| Clinical Stage | I | 24 (60.0) | 16 (40.0) | <0.001 |
|  | II | 72 (44.2) | 91 (55.8) |  |
|  | III | 25 (25.3) | 74 (74.7) |  |
|  | IV | 10 (26.3) | 28 (73.7) |  |
| T classification | T1 | 40 (54.1) | 34 (45.9) | 0.019 |
|  | T2 | 60 (34.9) | 112 (65.1) |  |
|  | T3 | 21 (31.3) | 46 (68.7) |  |
|  | T4 | 10 (37.0) | 17 (63.0) |  |
| N classification | N0 | 63 (43.4) | 82 (56.6) | 0.419 |
|  | N1 | 45 (36.0) | 80 (64.0) |  |
|  | N2 | 18 (32.1) | 38 (67.9) |  |
|  | N3 | 5 (35.7) | 9 (64.3) |  |
| M classification | No | 122 (40.4) | 180 (59.6) | 0.046 |
|  | Yes | 9 (23.7) | 29 (76.3) |  |
| Tumor grade | Well differentiated | 50 (55.6) | 40 (44.4) | 0.089 |
|  | Moderately differentiated | 36 (26.7) | 99 (73.3) |  |
|  | Poorly differentiated | 35 (43.8) | 45 (56.2) |  |
|  | Unknown | 10 (28.6) | 25 (71.4) |  |
| Molecular subtype | Luminal A | 45 (56.3) | 35 (43.2) | 0.078 |
|  | Luminal B | 21 (20.0) | 84 (80.0) |  |
|  | TNBC | 37 (41.1) | 53 (58.9) |  |
|  | HER2(+) | 28 (43.1) | 37 (56.9) |  |
| Relapse | Yes | 32 (24.4) | 93 (44.4) | 0.001 |
|  | No | 99 (75.6) | 116(55.6) |  |

Table S3

Univariate and multivariate analyses of various prognostic parameters in patients with breast cancer Cox-regression analysis

| Factor | Univariate analysis |  | Multivariate analysis |  |
| --- | --- | --- | --- | --- |
|  | HR (95% CI) | *P*-Value | HR (95% CI) | *P*-Value |
| Age | 1.221(0.778-1.541) | 0.142 |  |  |
| Clinical staging | 1.587(0.512-2.013) | 0.009 | 1.451(0.213-2.196) | 0.029 |
| T classification | 1.823(0.901-2.811) | 0.041 | 1.539(0.812-2.451) | 0.101 |
| N classification | 2.191(1.301-3.012) | <0.001 | 1.703(1.349-2.149) | 0.001 |
| M classification | 1.957(1.212-2.871) | <0.001 | 2.389(1.802-3.168) | 0.021 |
| Molecular subtype | 2.134(1.198-3.171) | 0.002 | 1.205(1.087-1.335) | 0.040 |
| Tumor grade | 1.319(0.598-2.156) | 0.031 | 1.019(0.276-1.981) | 0.053 |
| Relapse | 1.671(1.116-2.785) | 0.044 | 1.319(0.387-2.017) | 0.109 |
| TRIP6 expression | 2.331(1.337-3.012) | 0.011 | 3.131(2.154-4.215) | 0.038 |
